# Effect of molecular weight of hyaluronic acid (HA) on viscoelasticity and particle texturing feel of HA dermal biphasic fillers

**DOI:** 10.1186/s40824-016-0073-3

**Published:** 2016-09-07

**Authors:** Cheolbyong Chun, Deuk Yong Lee, Jin-Tae Kim, Mi-Kyung Kwon, Young-Zu Kim, Seok-Soon Kim

**Affiliations:** 1 Department of Biomedical Engineering, Daelim University, Anyang, 13916 Republic of Korea; 2 R&D Center, Neobiotech Co., Ltd, Seoul, 152-789 Republic of Korea; 3 Vericom Co., Ltd, Anyang, 606-72 Republic of Korea; 4 Beautiful Revolution Co., Ltd., Seoul, 135-513 Republic of Korea

**Keywords:** Dermal biphasic filler, Hyaluronic acid, Microsphere, Elastic modulus, Particle texturing feel

## Abstract

**Background:**

Hyaluronic acid (HA) dermal biphasic fillers are synthesized for their efficacy in correcting aesthetic defects such as wrinkles, scars and facial contouring defects. The fillers consist of crosslinked HA microspheres suspended in a noncrosslinked HA. To extend the duration of HAs within the dermis and obtain the particle texturing feel, HAs are crosslinked to obtain the suitable mechanical properties.

**Results:**

Hyaluronic acid (HA) dermal biphasic fillers are prepared by mixing the crosslinked HA microspheres and the noncrosslinked HAs. The elastic modulus of the fillers increased with raising the volume fraction of the microspheres. The mechanical properties and the particle texturing feel of the fillers made from crosslinked HA (1058 kDa) microspheres suspended in noncrosslinked HA (1368 kDa) are successfully achieved, which are adequate for the fillers.

**Conclusions:**

Dermal biphasic HA fillers made from 1058 kDa exhibit suitable elastic moduli (211 to 420 Pa) and particle texturing feel (scale 7 ~ 9).

## Background

In its natural state, hyaluronic acid (HA) exhibits poor biomechanical properties as a dermal fillers due to the poor viscoelasticity and the short half-life of HA when injected into normal skin [1–11]. To provide the ability to lift and fill wrinkles in the skin, chemical modification is required to improve its mechanical properties. Crosslinking is attempted to improve biomechanical properties while maintaining biocompatibility and biological activity [1–11]. HA dermal fillers are classified into two types, monophasic and biphasic [3–8]. Monophasic HA fillers (Surgiderm 24XP, England, Juvederm Ultra™, USA) are known as solely crosslinked and non-particulate HA gels [3, 4]. The monophasic fillers are prepared by mixing the high-molecular-weight HA and the low-molecular-weight HA. They have weak strength of gel and can easily transform by external force. No appreciable particle texturing can be felt when touching gels with hands. In contrast, biphasic fillers (Restylane®, Q-Med, Sweden) consist of gel particles of stabilized HA suspended in a noncrosslinked HA (NHA) [3, 4]. The crosslinked HA (CHA) gel particles are suspended in NHA, which acts as a lubricant, allowing the suspension to be pushed through a fine needle [4]. They are reported to have a rapid initial degradation of NHA and a slower degradation of the crosslinked gel particles, whereas monophasic gels are degraded more uniformly [3–5]. Restylane® is a FDA-approved dermal filler made of a biodegradable, non-animal stabilized HA. However, no biphasic fillers having suitable viscoelasticity and particle texturing feel (PTF) were made in Korea to replace the Restylane fillers. Therefore, the development of the domestic biphasic fillers is necessary.

Restylane® products (biphasic gel) are the most widely used fillers in the market. The ratio of CHA to NHA is 75:25, which is attributed to the restoration of facial volume [7]. Crosslinked gel nanoparticles are previously studied to feel the particle texture. Nanoparticles are synthesized after the consumption of carboxyl groups by crosslinker of adipic acid dihydrazide [7–11]. The nanoparticle size decreases from 140 nm to 95 nm with increasing the HA molecular weight (MW) from 697 kDa to 1368 kDa [12]. The decrease in nanoparticle size with increasing the MW may be due to intramolecular crosslinking rather than intermolecular crosslinking [9]. However, the flowability of nanoparticles rises dramatically due to the loss of viscous modulus, which is detrimental to dermal fillers. The elastic modulus (G’) is determined to be 178 Pa for the fillers composed of 15 % crosslinked nanoparticles suspended in 85 % of NHA [12]. Although the elastic modulus is similar to that of Restylane® (175 Pa), the amount of the CHA decreases dramatically from 85 to 15 %, which is detrimental to facial volume and fullness to the skin as dermal fillers. The 15 % portion is also very low as compared to those (60 ~ 70 %) of monophasic fillers [12, 13]. In addition, PTF is not achieved probably due to poor elasticity of nanoparticles [13].

HA dermal fillers having different ratios (65/35 to 95/5) of CHA microspheres (CHMs, 1058 kDa) to NHAs are synthesized to investigate the effect of CHMs on the variation of viscoelasticity and PTF [13]. The diameters of CHMs are in the range of 60 to 100 ± 4 μm with a 3-D porous structure channeled with 2 to 4 ± 0.5 μm. The fillers consist of gel particles with 300 ± 30 μm in size. G’ increased from 211 Pa to 700 Pa with raising the volume fraction of CHM from 65 to 95. The fillers having the CHM ratios of 65 to 85 exhibit the G’ values in the range of 175 Pa to 430 Pa, which can be extruded through the 29 ~ 30-gage needle. Experimental results revealed that PTF rose with increasing the volume fraction of CHM due to high density of gel particles. Excellent gel injectability and PTF were successfully achieved. In the present study, HA dermal fillers were prepared by using CHMs made from different MWs (697 kDa and 1058 kDa) to investigate the effect of MW on viscoelastic properties of the fillers.

## Methods

### Materials

HA with different MWs (697, 1058 and 1368 kDa) was purchased from Shiseido Company (Tokyo, Japan). Divinyl sulfone (DVS, 97 %) was purchased from Sigma-Aldrich (Germany). 2-methyl-1-propanol (99 %), ethanol (99.5 %), and sodium hydroxide (bead, 98 %) were obtained from Samchun Pure Chemical Company (Korea).

### Microsphere preparation

HA solutions of 0.5 wt.% concentration were prepared by dissolving sodium hyaluronates (*Streptococcus*, Mw =697 and 1058 kDa, Shiseido Co., Japan) in 0.05 mol/L NaOH for 24 h at room temperature. A pH in the range of 11 to 12 was adjusted by adding 10 mol/L NaOH to the HA solution. Then, the HA solution was placed in a solution hopper attached to the Masterflex L/S tubing pump (Cole Parmer, USA) and fed into a syringe equipped with a 22-gage metal needle with a flat outlet at a flow rate of 0.005 mL/min [13–16]. The distance between the nozzle and the solution was 4 cm. Microspheres (MSs) were fabricated by supplying compressed air (0.034 MPa) along the HA solution nozzle. The nozzle was enclosed by a delivery tube with a diameter of 6 mm. MSs were collected in a solution mixture of 0.2 vol.% of DVS in 2-methyl-1-propanol, followed by a stirring process (MST Digital, IKA, Germany) for 24 h at RT. Then, the crosslinked MSs were screened through a 325 mesh sieve. The MSs were immersed in distilled water for 0.5 h and ethanol for 0.5 h to remove the residual crosslinker. After removal of an unreacted residual crosslinker, the MSs were then dried for 2 h at 60 °C in a vacuum of 20 Torr. The as-dried MSs were examined by using SEM (S-3000H, Hitachi, Japan) and optical microscopy (SV-55, Sometech, Korea) to investigate the morphology and the size of the MSs. Prior to the SEM measurements, all specimens were coated with Au/Pd to ensure higher conductivity.

### Hydrogels

The HA hydrogels (HAHs) are biphasic products consisting of CHMs (697 and 1058 kDa) suspended in NHA (1368 kDa) used as a carrier. For gel preparation, NHAs (20 mg/mL) dissolved in a phosphate buffered saline solution (PBS, NaH_2_PO_4_) and swollen CHMs were homogenized for 1 ~ 4 min and then incubated for 24 h [12, 13]. The ratio of CHA to NHA is varied from 65:35 to 95:5. The elastic and viscous response of hydrogel depend on the concentration and MW of the HA and on the frequency used during the measurements. Rheological behavior of HAHs were analyzed with a Thermo Haake RS1 Rheometer (Newington, USA), using a plate and plate geometry with a 1.2 mm gap [12, 13]. All measurements were performed using a 20 mm titanium sensor at 25 °C. Oscillation measurements were taken at 5 Pa tau over a frequency range of 0.01 to 100 Hz [13]. The measured properties of hydrogels was evaluated by measuring the elastic modulus (G’) and viscous modulus (G”). The measured modulus at frequency of 5 Hz for HAHs was compared [12]. In addition, PTF is evaluated by observing or touching the gels using an optical microscope (Pro Camscope, Sometech, Korea) or fingers, respectively.

### Swelling property

The swelling characteristics were measured by immersing weighed samples of dry MSs for 24 h in PBS. The excess surface water in the swollen gel was removed by blotting and then the swollen gel was weighed. After measuring the weight of the gels, the swelling ratio was determined [12, 13].

### Cytotoxicity

The extract test method was conducted on the HAHs to evaluate the potential of cytotoxicity on the base of the International Organization for Standardization (ISO 10993–5). The HAHs were extracted aseptically in single strength Minimum Essential Medium (1X MEM) with serum. The ratio of HAH to extraction vehicle was 4 g/20 mL. The test sample was used within 24 h after completion of the preparation. The test extract was placed onto three separate confluent monolayers of L-929 (NCTC Clone 929, ATCC, USA) mouse fibroblast cells propagated in 5 % CO_2_. For this test, confluent monolayer cells were trypsinized and seeded in 10 cm^2^ wells (35 mm dishes). Simultaneously, triplicates of reagent control, negative control (high density polyethylene film, RM-C), and positive control (polyurethane film, RM-A) were placed onto the confluent L-929 monolayers. All monolayers were incubated for 48 h at 37 °C in the presence of 5 % CO_2_. After incubation, the morphological change of the cell was examined to assess the biological reaction by using the inverted microscope (TS100-F, Nikon, Japan) and the iMark microplate absorbance spectrophotometer (Bio-Rad, USA). Water-soluble tetrazolium salts (WSTs) are a series of other water-soluble dyes for MTT assays, which can provide different absorption spectra of the formed formazans. EZ-cytox yields a water-soluble formazan, which can be read directly. The absorbance of the colored solution is quantified by measuring at a wavelength of 415 nm with the iMark microplate absorbance spectrophotometer. The value of untreated cell (control sample, only cultured with culture medium) was set as 100 % and those of the treated cells were expressed as the percentage of the control sample.

### Particle texturing feel (PTF)

It is hard to quantify the PTF using an optical microscope or fingers. Although touching gels with hands is very subjective, PTF is scaled qualitatively from 0 (lowest) to 10 (highest) to provide more understanding. When MSs were easily broken like gels and disappeared, PTF may be 0 ~ 2. PTF is determined to be 7 ~ 9 when MSs were remained as particles on fingers after rubbing repeatedly.

### Statistical analysis

All experiments were performed in triplicate. Values in the text are expressed as the means ± standard deviation (SD), and *p* < 0.05 was considered statistically significant.

## Results and discussion

### Morphology of crosslinked microspheres

Two types of CHMs having different MWs (697 and 1058 kDa) were fabricated [13–19]. The average diameter of CHMs (HA = 697 and 1058 kDa) was in the range of 158 ± 16 μm and 100 ± 4 μm and the swelling rate of 1270 and 1000 %, respectively. The MW of HA used for MS fabrication influenced the size of the MS. The MSs made from 697 kDa HA were larger than the MSs prepared from 1058 kDa probably due to the intramolecular crosslinking rather than the intermolecular crosslinking caused by the short length of 697 kDa HA [9].

**Fig. 1:**
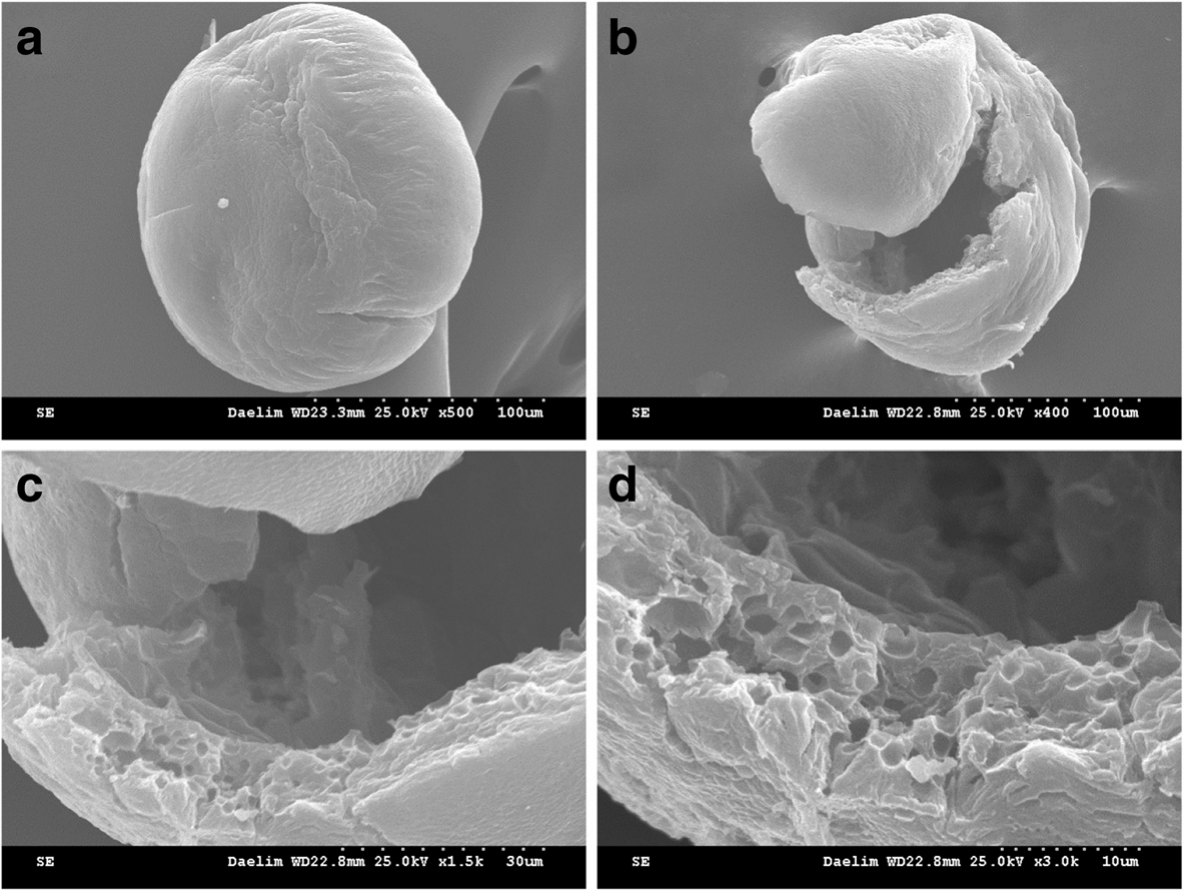
SEM images of (**a**–**d**) CHMs (HA = 697 kDa). Note that **b** ~ **d** images exhibit hollow structure inside the MS

**Fig. 2:**
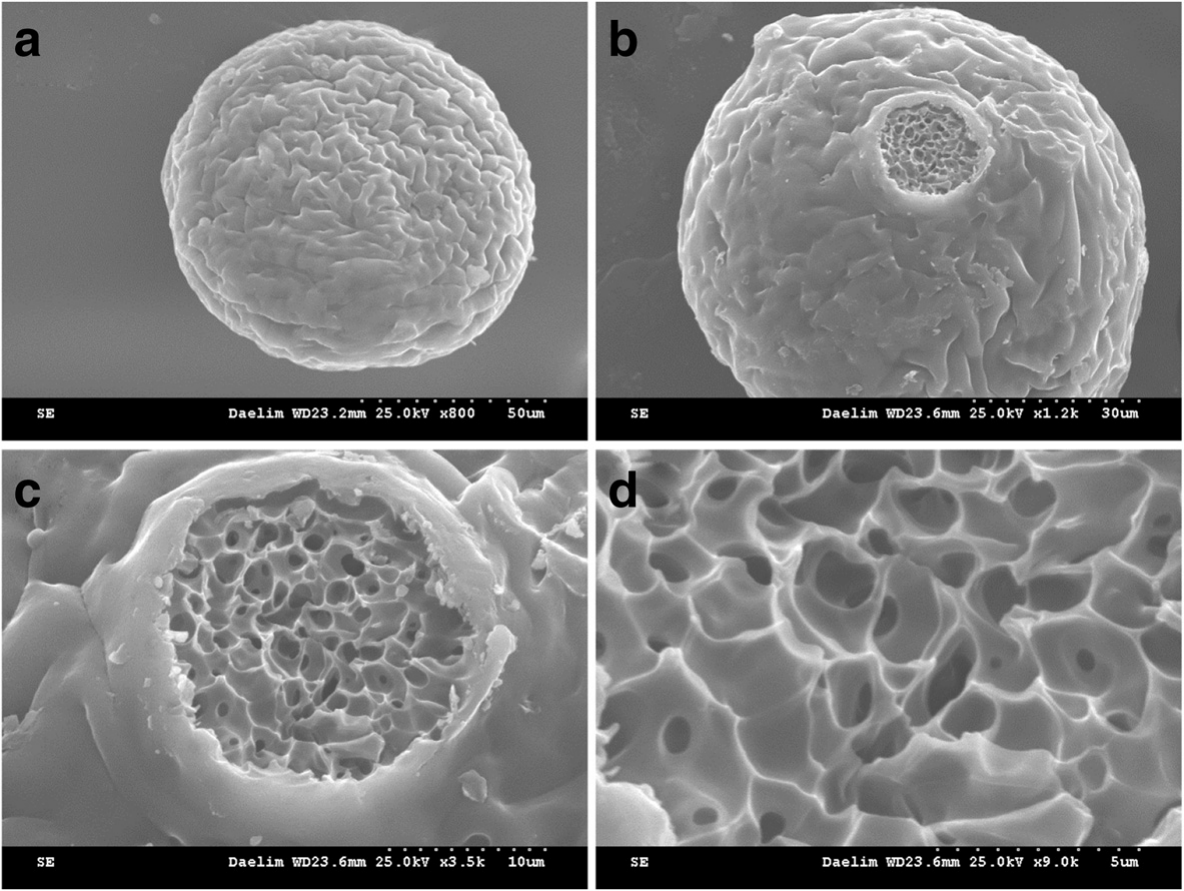
SEM images of (**a**–**d**) CHMs for 24 h. Note that **b** ~ **d** images exhibit porous network inside the MSs

Morphologically the MSs were white colored spheres having a smooth surface, as shown in Figs. 1 and 2. Hollow structure of the MSs made from 697 kDa HA was observed. The hollow MSs were an outerlayer of 10 ± 0.5 μm in thickness channeled with 1.7 ± 0.3 μm pores, which was indicative of poor particle compressibility (Fig. 1). In contrast, no pores on the surface of MSs made from 1058 kDa HA were visible. However, pores inside the MSs were easily seen (Fig. 2). The MSs were a 3-dimensional porous network structure channeled with 2 ~ 4 ± 0.5 μm pores, which represented higher particle compressibility [9].

### Biocompatibility

The HAHs were prepared by immersing the MSs in PBS solution by varying the ratio of CHA to NHA from 65:35 to 95:5. They can be used as the tissue membrane to release of entrapped drug under a certain condition in a controlled manner. It could be used to encapsulate and cultivate cells inside the gel, where the network will act as a semipermeable membrane allowing only growth factors to enter to aid the growth of cells [13]. MSs were crosslinked with DVS, followed by cleaning in ethanol and distilled water. Covalent linkages between polymer chains can be obtained by the reaction of functional groups of a DVS crosslinking agent (vinyl group) and HA (hydroxyl group) [11–24]. The presence of crosslinker’s residue in the HA hydrogels after cleaning was not detected after cleaning, suggesting that the unreacted residual crosslinker was successfully removed [12, 13].

**Fig. 3:**
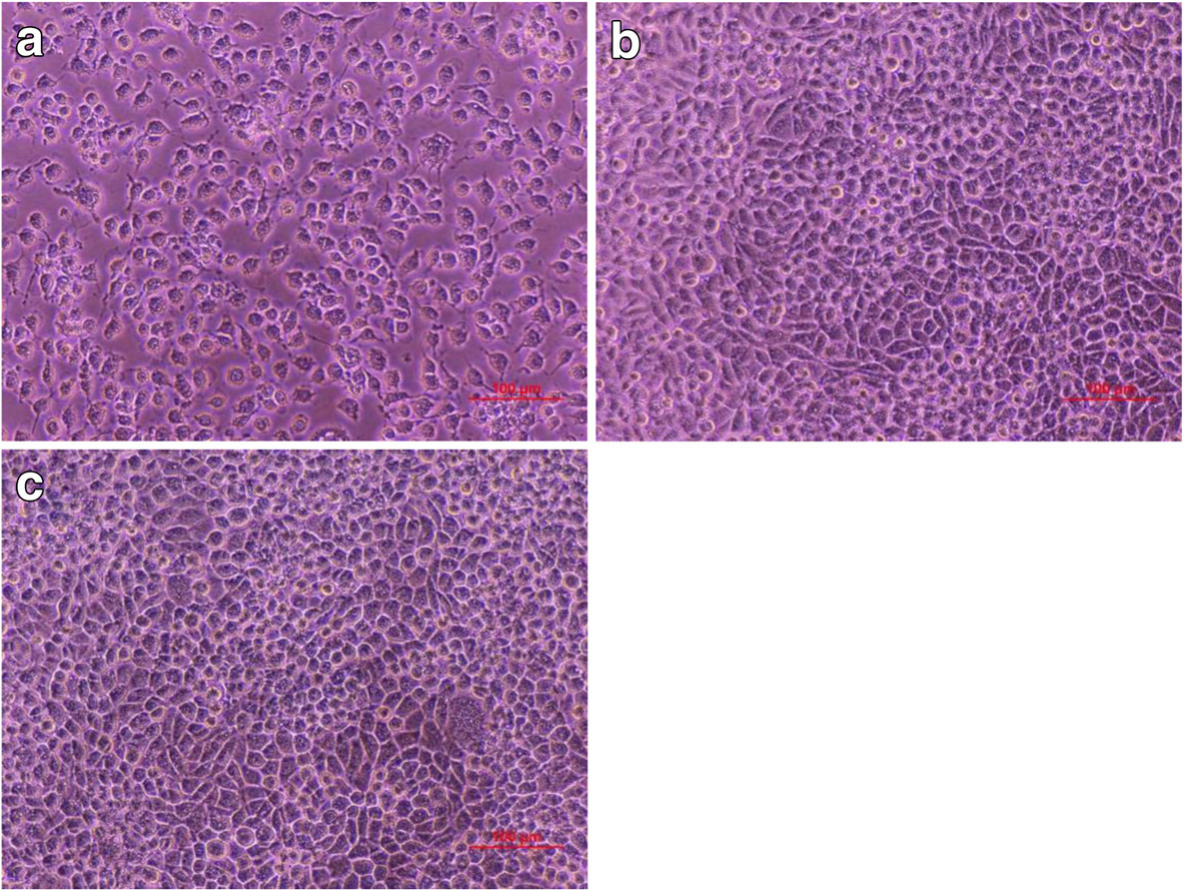
Photographs of cell morphology: **a** positive control, **b** negative control, and **c** the extract of HAHs from WST assay (EZ-cytox) after exposing with HAH suspensions for 48 h

A cytotoxicity test of the HAHs determines whether a product or compound will have a toxic effect on living cells. The test extract with the 75/25 ratio of CHA (1058 kDa) to NHA (1368 kDa) showed no evidence of causing cell lysis or toxicity, as depicted in Fig. 3. The HAHs exhibited equal or higher average cell amount (100.6 %) compared to the negative control, as verified by the microscope and the absorbance spectrophotometer. The qualitative morphological grading of cytotoxicity of extracts was determined to be scale 0.

The biocompatibilities of HA hydrogels, such as genotoxicity (in vitro chromosome aberration test, reverse mutation assay, in vivo micronucleus test), skin sensitization, intradermal reactivity, and pyrogenicity, were previously evaluated and determined to be suitable for soft tissue augmentation due to the absence of abnormal clinical signs [14, 15]. There was no difference between the HAHs and negative control mean scores because skin reaction such as erythema or oedema was not observed after injection. In addition, the HAHs had no subchronic systemic toxicity, indicating that the implants were excellent in biological synthesis and transplantation as evidenced by non-capsule reaction and disappearance of inflammatory cells [16]. It was reported that the implants of HAHs are clinically safe and effective.

### Viscoelastic property

Since the filling capacity of a dermal filler is known to be dependent on its water uptake capacity, the swelling ratio was examined. It is suggested that the highest water uptake indicates a lower crosslinking extent with respect to the other gels. On the contrary, the lowest swelling capacity may be due to a higher crosslinking density [12, 13]. It was evident that the expansion capacity of HA hydrogels rose with increasing the amount of NHA and was inversely proportional to the crosslinking degree [12, 13, 18]. The swelling ratios of NHA having the MW of 1368 kDa and the CHMs made from MW of 697 and 1058 kDa were 4000, 1260, and 1000 %, respectively [12, 13]. The swelling ratio of NHA increases with increasing MW. However, the swelling ratio of CHMs decreases with increasing MW probably due to an increased number of coiled HA chain interactions [12–17]. Because of short length of 697 kDa HA, the chances of intramolecular crosslinking occurring during the fabrication of MSs was expected to be higher than those of intermolecular crosslinking [9].

The ratios of CHA to NHA for commercially available dermal fillers are 98:2 for Hylaform/Prevelle, 75:25 for Restylane/Perlane, 60:40 for Juvederm (30 HV), respectively. In the present study, MSs made from 697 kDa and 1058 kDa HA were blended with 1368 kDa NHA. G’ of HA hydrogels was evaluated as a function of the volume fraction of CHA to NHA in the range of 65:35 to 95:5. Among the commercial fillers, Restylane is biphasic products consisting of CHM (75 %) suspended in NHA (25 %) used as a carrier.

The filling capacity is also affected by the surrounding tissue resistance to gel enlargement and by the rheological behavior of the filler itself during in situ implantation. To achieve correction of lines and wrinkles and restore volume, the gel implant should lift the tissue. The strong gel can provide the force required to lift the tissue and resist subsequent deformation, resulting in the desired correction. A high lifting capacity therefore requires high gel strength. The elastic modulus represents the stiffness of the gels and the ease of extrusion of the product. A 3-dimensional network of HAHs is easily formed when crosslinks between the HA chains are introduced. A strong gel has a high elasticity, meaning that the response to deformation is mainly elastic [2–4].

**Fig. 4:**
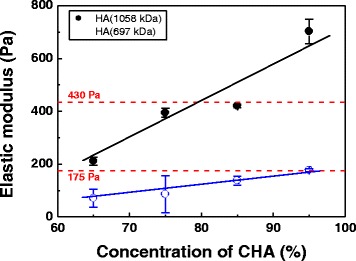
Elastic modulus as a function of concentration of CHA. Note that the region of the dotted lines represents the easy delivery of the fillers through a 29 ~ 30 gage needle

It is known that more deformable gels, having lower G’ values ranging from 120 Pa to 430 Pa, can be injected through the finer needles between 29 and 30 gage [6]. In contrast, larger needles (26 ~ 27 gage) are required for the extrusion of firmer gels (1400 ~ 1800 Pa). The G’ value of commercial soft skin filler, Restylane Skinbooster, is determined to be 175 Pa [12]. The G’ values of HA fillers having different ratios of DVS CHA to NHA are shown in Fig. 4. The G’ values increased from 71 ~ 177 Pa to 211 ~ 702 Pa with increasing the HA MW from 697 kDa to 1058 kDa. The fillers having the ratio of the CHA (697 kDa) to the NHA between 85:25 and 95:5 showed the G’ values in the range of 137 Pa to 177 Pa. The low G’ values of the fillers made from 697 kDa may be due to hollow structure of MS as a result of intramolecular crosslinking [19]. The G’ value of 177 Pa is observed only for the fillers containing 95 % of CHA MS, which is similar to that of Restylane products (175 Pa). Rest of the fillers made from 697 kDa HA do not meet G’ values of the commercial fillers, as displayed in Fig. 4. On the other hand, the fillers having the ratio of the CHA (1058 kDa) to the NHA between 65:35 and 85:25 showed the G’ values in the range of 175 Pa to 430 Pa [13]. They can be extruded through the 29 ~ 30-gage needle allowing for ease of injection, which can be applicable to the treatment of non-severe defects and generally for thinner and softer skin rather than the correction of deep folds and skin deformation [4]. The content of CHM is in the range of 65 to 85, which is similar to Restylane products widely used in the market [7, 13, 24]. The elastic moduli of the as-prepared fillers (211 ~ 420 Pa) are even stiffer than that of Restylane products (175 Pa) [13].

### PTF

**Fig. 5:**
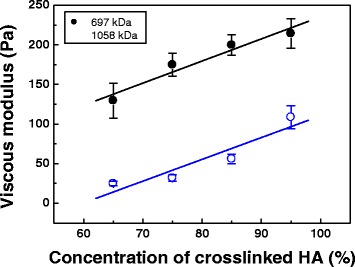
Viscous modulus as a function of concentration of CHAs made from 697 kDa and 1058 kDa HA

Unlike the monophasic fillers, the commercial Restylane fillers show PTF when touching gels with hands. The biphasic fillers prepared in the present study consist of crosslinked micro-sized gel particles suspended in NHA. Our previous studies revealed that the crosslinked nanoparticles of the dermal fillers increased the flowability dramatically, which was detrimental to PTF (scale 0) [12]. The nanoparticles contain a less-accessible crosslinked polymer structure, thus reducing polymer chain interactions and thereby lowering the viscosity. Viscous modulus (G”) is also called loss modulus because it describes the energy that is lost as viscous dissipation. The G” value is a measure of the flow (rheological) properties for the fillers. The G” values as a function of concentration of crosslinked HA are shown in Fig. 5. Appreciable difference in G” is found. The G” values of the fillers made from 697 kDa are in the range of 25 to 108 Pa, which is lower than those of commercial products. The PTF scale is likely to be in the range of 1 to 2. However, the fillers made from 1058 kDa show higher values than 128 Pa, which correspond to the PTF scale in the range of 7 to 9. The G” values of the as-prepared fillers (129 to 214 Pa) are better than those of Restylane (119 Pa) and Perlane (125 Pa), which are categorized in the medium-viscosity and –elasticity group [25, 26].

**Fig. 6:**
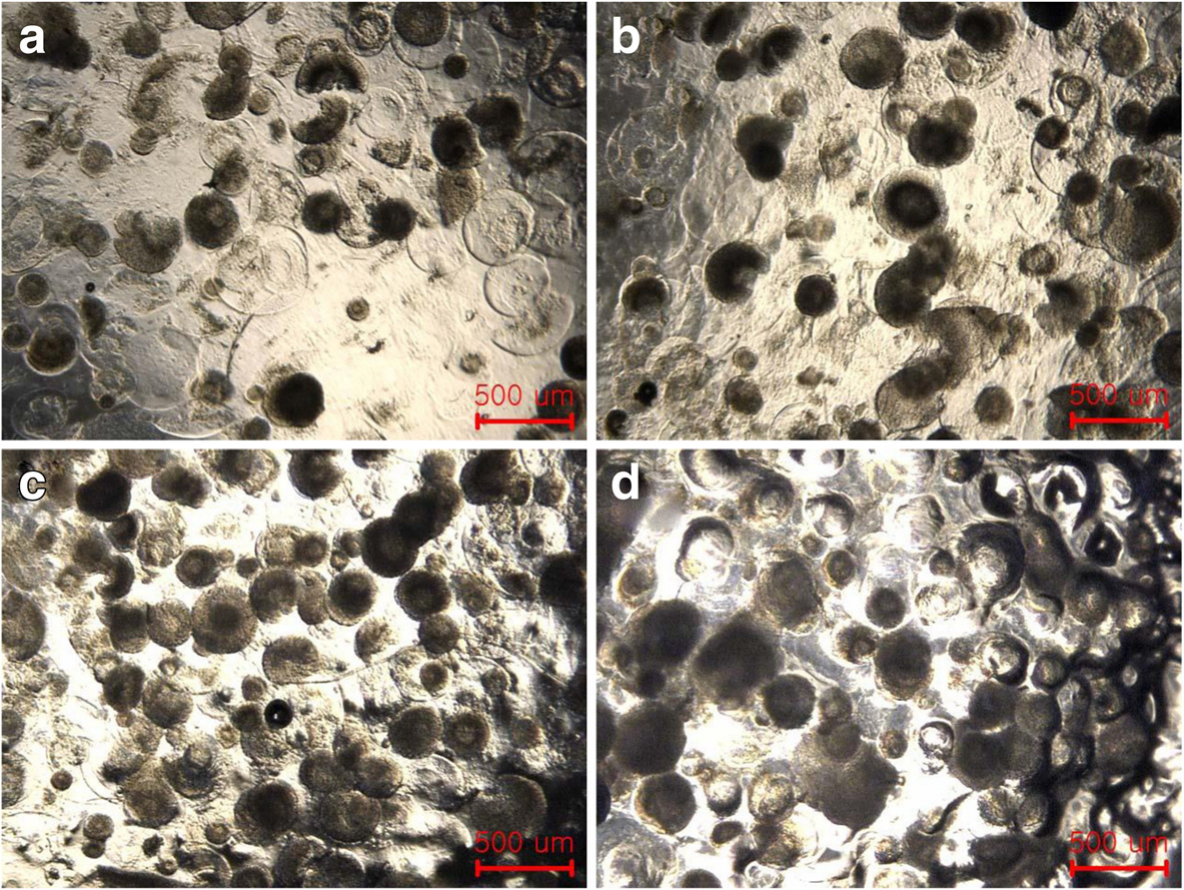
Optical photographs of the fillers having the volume fraction of CHMs (Mw = 697 kDa): **a** 65 %, **b** 75 %, **c** 85 %, and **d** 95 %, respectively

PTF is evaluated qualitatively by observing or touching the gels using an optical microscope or fingers due to the presence of crosslinked gel particles, respectively. No appreciable difference of CHM images can be found (Fig. 6 and reference 13). The swollen particle size made from 697 kDa and 1058 kDa HA is 260 ± 48 μm and 300 ± 30 μm, respectively. Although no flowability is detected for both fillers, PTF of fillers made from 697 kDa HA is not tangible due to poor compressibility of MSs, as verified by SEM observation and viscoelastic properties. However, excellent PTF is observed for the fillers made from 1058 kDa. As the volume fraction of CMSs increased, the particle density rose as expected. It can be concluded that the dermal biphasic fillers possessing the tailored elasticity and PTF are successfully achieved for the fillers made from 1058 kDa HA.

## Conclusions

Viscoelasticity of HA dermal fillers, made from 697 kDa and 1058 kDa HA suspended in NHA (1368 kDa), is examined by using the SEM, the optical microscope, and the rheometer. The average diameter of CHMs made from 697 kDa and 1058 kDa HA is 158 ± 16 μm and 100 ± 4 μm and the swelling ratio of 1270, 1000 %, respectively. Unlike the fillers made from 697 kDa HA, the fillers (1058 kDa) exhibit tailored elastic moduli (211 ~ 420 Pa) and viscous moduli (129 ~ 214 Pa), which are adequate for dermal biphasic fillers. PTF (scale 7 ~ 9) is also successfully achieved due to excellent viscoelasticity.
